# Supplementary material for: First Record of Microplastic Contamination in the Non-Native Dark False Mussel Mytilopsis leucophaeata (Bivalvia: Dreissenidae) in a Coastal Urban Lagoon
Source: Int J Environ Res Public Health. 2023 Dec 27;21(1):44. doi: 10.3390/ijerph21010044 (PMC10815431; doi:10.3390/ijerph21010044)
Supplement: Supplementary file 1 [file ijerph-21-00044-s001.zip › Table S1.pdf]

Table S1. Water temperature and salinity in the ten sampling areas at Rodrigo de Freitas Lagoon (Rio de Janeiro, Brazil).

Sampling area	Temperature (ºC)	Salinity (ppt)
A1	12.95	28.90
A2	13.65	29.35
A3	13.80	28.90
A4	12.95	28.90
A5	12.10	29.35
A6	12.80	28.80
A7	13.15	29.00
A8	12.80	31.75
A9	12.90	30.55
A10	12.90	30.20
