# Supplementary figures and images for: First Record of Microplastic Contamination in the Non-Native Dark False Mussel Mytilopsis leucophaeata (Bivalvia: Dreissenidae) in a Coastal Urban Lagoon
Source: Int J Environ Res Public Health. 2023 Dec 27;21(1):44. doi: 10.3390/ijerph21010044 (PMC10815431; doi:10.3390/ijerph21010044)

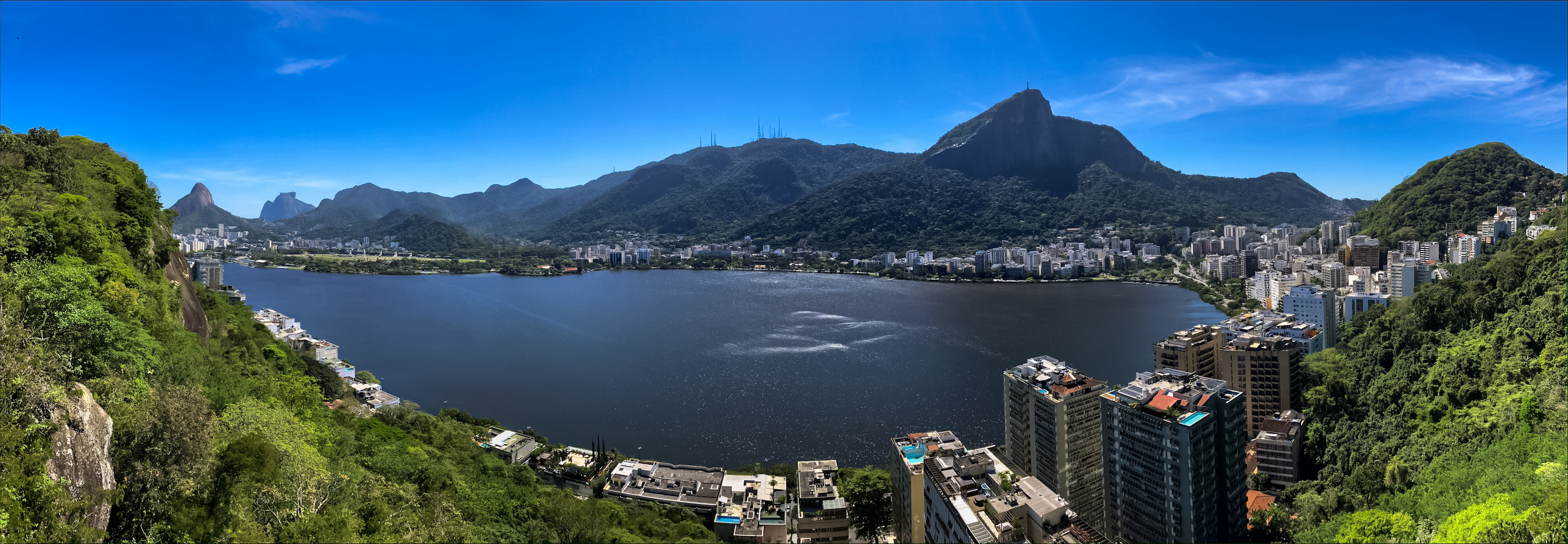

Supplement: Supplementary file 1 [file ijerph-21-00044-s001.zip › Figure S1.jpeg]
